# Use of WHO Growth Standards Rather Than Locally Specific Linear Growth Curves Promotes Equity in Pediatric Growth Research for Children Younger Than 5 Years

**DOI:** 10.1093/nutrit/nuaf091

**Published:** 2025-06-16

**Authors:** Amelia B Finaret, Precious Taylor-Forde

**Affiliations:** Department of Global Health, Allegheny College, Meadville, PA, United States; Global Agriculture and Food Systems, University of Edinburgh, Edinburgh, United Kingdom; Department of Global Health, Allegheny College, Meadville, PA, United States; Brown University, Providence, RI, United States

**Keywords:** linear growth, stunting, WHO growth curves, growth standards, growth references, race, ethnicity, nationality, children

## Abstract

There is strong evidence that healthy children around the world grow according to the World Health Organization Child Growth Standards when they benefit from healthy environments, regardless of race, ethnicity, or nationality. Despite this, arguments still exist in the scientific literature that child growth curves specific to local populations are necessary. We use a narrative review of the literature on child growth to focus on articles in which different, locally specific child growth curves have been developed or recommended. We synthesize the arguments against a universal child growth standard to provide an understanding of these problematic claims, in the context of new efforts to address remaining echoes of scientific racism in the field of nutrition and other biomedical sciences. Child nutrition assessment should take place using high-quality tools and metrics that do not depend on race, ethnicity, or nationality.

## INTRODUCTION

Growth references describe the heights, weights, and other anthropometric characteristics, like head circumference, by age and sex of a group of children over time. The linear growth of children is the single best marker of child nutritional status in that it is associated with adverse health and socioeconomic outcomes. Linear growth faltering is tracked against global growth standards, which describe how healthy children should grow.^1^ The most easily observed (ie, without laboratory tests or a clinical exam) disparities in nutritional status between groups of children, such as for height-for-age, may create an illusion that children grow differently depending on their race or ethnicity. However, there is not a biological basis for race or ethnicity, and researchers should not model racial or ethnic categories as determinants of nutrition.^2^^,^^3^ The recent report from the National Academies of Sciences argues that using race or ethnicity as shortcuts for investigating the determinants of health or disease can prevent researchers from gaining deeper understanding, such as by using high-quality genetic or environmental data.^3^

The articles cited in this review, found particularly in the public health, human biology, and physical anthropology literatures, argue for various reasons that locally specific growth curves are necessary as opposed to the World Health Organization (WHO) Standard Child Growth Curves. We summarize some of these articles and then describe specific opportunities to improve equity in research on child growth. By equity in child growth research, we contend that child nutrition assessment should take place using high-quality tools and metrics that do not depend on race, ethnicity, or nationality.^3^ Although the WHO standard child growth curves have been widely adopted since 2007, the number of current studies that argue for locally specific growth curves, specific to race, ethnicity, or nationality, is concerning.

For clarity in the context of this article, our definitions are that race divides people into groups based on physical characteristics such as skin color, whereas ethnicity refers to the cultural identification for people who share common geographies, languages, traditions, or histories. Both concepts are social constructs, and both concepts can lead to discrimination and inequity due to perceived differences between groups of people and the notions of race and ethnicity as intrinsic or essentialized, especially when compared with other notions of human existence.^3^

## METHODS

A literature search was conducted without a restriction on publication date using the PubMed, Web of Science, and ProQuest Public Health databases, using the following key terms: race, ethnicity, nationality, “local population” and misclassification; combined with terms about growth curves including “growth references,” “growth standards,” “growth curves,” “WHO child growth standards,” and “adoption of growth standards”; combined with terms about child health and nutrition, including stunting, “linear growth,” and height. We also conducted hand searches of the references lists of articles that met search criteria. Articles were selected on the basis of whether they argued that any given population should not use the WHO Child Growth Standards because there are or may be racial or ethnic differences in height attainment.

## RACE AND ETHNICITY ARE NOT DETERMINANTS OF CHILD GROWTH

Decades before the WHO Child Growth Standards (henceforth, WHO Standards) were developed, some prescient scholars argued that racial or ethnic differences in child growth should not be considered, because they are small, if they exist at all, compared with socioeconomic or environmental differences.^4^ Patterns of human growth vary widely across and within populations, and across generations as well.^5–7^ As far back as the early 20th century, anthropologist Frans Boas argued that the environment is more important than genetic ancestry in determining body size and shape.^8^ Since then, a wide-ranging literature examining the determinants of stunting has found, for example, associations with vitamin A deficiency,^9^ mycotoxin exposure as a potential causal factor,^10^ and environmental enteric dysfunction as a potential causal factor.^11^

Instead of modeling racial or ethnic categories as determinants of nutrition, researchers should work to incorporate the social determinants of health in quantitative and qualitative analyses, including the consequences of experienced racism, discrimination, and bigotry.^2^ Between 1995 and 2018, the uses of race or ethnicity as quantitative variables in epidemiological research have not improved, unfortunately, with only 4 of 1050 reviewed articles even defining race or ethnicity.^12^ In addition, most studies did not explain how race or ethnicity were measured, and most studies simply included race or ethnicity as control variables without sufficient elaboration.^12^

Whereas most social scientists and nutrition scholars conceptualize race as nonbiological social constructs, current biomedical approaches to genetic ancestry have upheld the ideas of delineated races and ethnicities and of race or ethnicity as intrinsic.^3^^,^^13^ The delineations of racial or ethnic groups were a guiding paradigm for anthropology, anthropometry, and for statistics in the early beginnings as scientific fields. Echoes of racial and ethnic delineations remain, including that different sets of growth curves for local populations have been developed and recommended for use instead of the WHO Standards. In this article, we review the remaining echoes of racial and ethnic delineations in child growth research, focusing on articles published since the WHO Standards were released. Through this review, we aim to support nutrition researchers and clinicians to enhance equity in child growth research by refuting claims that locally specific growth curves are necessary.

## BACKGROUND ON THE DEVELOPMENT OF THE CHILD GROWTH STANDARD

The WHO Multicentre Growth Reference Study (MGRS), which took place between 1997 and 2003 and enrolled children from Brazil, Ghana, India, Norway, Oman, and the United States, demonstrated that groups of children around the world grow similarly when they are not exposed to adverse nutritional environments.^14^ All participants in the MGRS study met the same predetermined criteria, including 1) an absence on environmental or socioeconomic constraints on growth; 2) adherence to infant and young child feeding recommendations including breastfeeding; 3) singleton birth; 4) healthy with no significant morbidities; and 5) and born to mothers who did not smoke. Race or ethnicity were not part of the criteria for inclusion in the study.

The WHO Child Growth Standards, which were developed from the MGRS, describe how children everywhere should grow if they benefit from healthy environments.^1^ The International Fetal and Newborn Growth Consortium for the 21st Century has since developed standards from 9 weeks of gestation to age 5 years, finding yet again that when children benefit from a healthy environment, the main source of variation in their growth is individual variation, not ethnic variation.^15^

Many researchers and 140 countries have adopted the WHO Standards since their publication, and the MGRS findings are still a comprehensive and high-quality source of information about how healthy children grow.^1^^,^^16^ Country-level adoption of the WHO Standards was arguably widespread even though using the new standard worsened the prevalence estimates of various forms of malnutrition estimated at the country level.^17^

Similar data on child growth for school-age children (≥5 years of age) and adolescents do not exist.^18^ Although there is a WHO growth reference for children aged 5–19 years, it was not possible to develop a growth standard for school-age children and adolescents, because measuring all the environmental and social factors that determine growth for older children over long periods is too complex and because there is a wider range of normal growth as children age.^18^^,^^19^ Given these complicating factors, this review focuses on the evidence for infants and children <5 years of age for whom a growth standard does exist.

Together, a child’s genes, environment, and various gene-environment interactions determine linear growth and can even affect a child’s phenotype starting not only with fetal exposures but also with parent and grandparent exposures.^7^ A lack of access to food, malabsorption, micronutrient deficiencies, chronic inflammation, repeated infection, and chronic sleep deprivation can all contribute to poor growth outcomes.^20^ Child growth is multifactorial and has wide interindividual variability, so one may question whether it is acceptable to generate standards for human body sizes at all, especially when attained child heights are often incorrectly made synonymous with poor health.^21^^,^^22^

When trying to define a health standard, such as for child growth, there are technical challenges and political challenges. Public health officials and government stakeholders may have preferences for how healthy child growth should be defined in their context, and they might be concerned that citizens of their diverse populations might not be appropriately represented in a universal standard. Implementing a health standard could also exacerbate disparities or interact with politics and power in a country, especially as countries are aiming for targets of the Sustainable Development Goals (SDGs).^17^^,^^23^ As of 2022, the United Nations estimates that 148 million children <5 years old around the world have stunting.^24^ In the context of monitoring progress toward the SDGs, UNICEF has developed guidance for countries on estimating stunting, stating that “estimates may not be comparable if they are based on different reference populations or children of different ages”^25^ and that if a country uses a different reference population for data reporting on child growth, UNICEF will recalculate those data to be based on the WHO Standards.^25^

Finally, using the standards or references themselves is subject to errors, such as technical errors when measuring individual children, confirmation bias or priming based on prior beliefs of survey enumerators, heaping on height measurements and on age, and nonrandom missing data.^25^^,^^26^ Despite these challenges, height measured against a standard can capture a wide range of insults to human health, and understanding shifts in the distribution of heights over time is a “productively simplifying” exercise for research and for policymaking.^27^

## OPPORTUNITIES FOR IMPROVING EQUITY IN CHILD GROWTH RESEARCH

To enhance equity by avoiding racial essentialism in the child growth literature across disciplines, there are three areas that nutrition researchers can focus on. First, nutrition researchers should communicate in their writing and outreach that comprehensive statistical modeling of the determinants of child growth is challenging, and causal inference is only possible with certain study designs. Second, nutrition researchers can disentangle the clinical vs epidemiological meanings of growth faltering in their work. Finally, nutrition researchers can distinguish in their writing and outreach between growth standards and growth references. The following sections outline these three opportunities while summarizing the articles that argue for locally specific growth curves.

### Comprehensive Statistical Modeling of the Determinants of Child Growth Is Challenging

Here, we describe the statistical and data-related challenges with modeling child growth, and summarize articles that argue for locally specific child growth curves because all possible determinants of linear growth were “controlled for.” In claiming that all determinants of child growth were controlled for, some articles argue that the only remaining explanations for difference between groups of children are population genetics, genetic ancestry, ethnicity, or race. Because the process of attained height convergence at the population level is long term and involves intergenerational mechanisms, it may appear that height attainment depends on ethnicity or race, but this is an illusion. Growth faltering is a long-term adaptive process, including tradeoffs that preserve brain size to the detriment of leg length.^22^^,^^27–29^

Several important sources of residual confounding or mediation in models where height-for-age is the outcome include 1) diarrheal disease or other infection^1^^,^^30^; 2) intergenerational factors^31–33^; 3) inequality in access to health care^18^; 4) intrahousehold effects^18^; 5) fetal growth restriction^20^; and 6) socioeconomic inequality.^34–36^ Even with robust measurements and study design, it would be impossible to effectively control for all the determinants of linear growth. Adjusting for maternal height to prevent “overestimating” child stunting in India, as recommended by Subramanian et al,^37^ is impossible and unethical because maternal height is not exogenous to many modifiable factors that also determine child stature and other important child health outcomes that relate to stature.^37^^,^^38^ If child growth differs based on race or ethnicity due to the challenge of statistical modeling of observational data, there is a significant risk of setting lower expectations for child growth for children who are marginalized by poverty.^38^

Some examples in the literature that claim to comprehensively model the determinants of growth include articles by Blackwell et al^39^ and Martin et al,^40^ who argued that choice of a local reference vs the WHO standard for the Indigenous South American Tsimane people affects the estimated relationships between linear growth and the determinants of linear growth. But their Tsimane-derived reference shows 0% of children as stunted, whereas the WHO Standard estimates that 23 (*N* = 156) of included children had stunting. The authors stated correctly that environmental factors matter for disparities in Tsimane child growth, but they still analyzed Tsimane children using a locally specific reference curve despite the highly adverse conditions in which Tsimane children grow, including a 50% prevalence of hookworm infection.^39^^,^^40^ Zong and Li^41^ argued that the differences found between the WHO Standards and a reference population of children in China are mainly due to “different ethnic backgrounds,”^41^ despite also claiming that the height increases for Chinese children are attributable to economic development. Park et al^42^ found that Canadian children were longer and taller than the WHO Standards across the distribution of height-for-age z-score (HAZ), and the authors suggest an analysis by parental ethnicity to understand why.

Rojroongwasinkul et al^43^ described that not all the exclusion criteria for the MGRS were used for their sample, including the inability to detect anemia, adverse birth outcomes, breastfeeding, or family income, but they still claim that, for children in Southeast Asia, South-East Asian Nutrition Surveys-derived curves are preferable to use to the WHO standards. Similarly, Hui et al^44^ noted that “epigenetic constraints on growth,” which were not measured in their study, may have caused the disparities between their sample in Hong Kong and the WHO Standards, but they still argued that the WHO Standards may not be appropriate for all.

Tarozzi et al^45^ find strong evidence that children in families who migrate to higher-income countries experience a decrease in nutritional deficits over time, but the authors still argued that the results could not be taken as conclusive in case the families who migrated are “genetically different” from those who stayed behind. In another example of studying people who have migrated, de Wilde et al^46^ claimed that children of South Asian descent living in the Netherlands are shorter than White children, due to ethnic differences, even though the authors did not control for various socioeconomic factors.

Race and ethnicity as variables in a statistical model are not easily defined or measured, because their social construction changes over time.^2^^,^^3^ Race and ethnicity often explain much of the variation in many health outcomes because race and ethnicity are associated with many different social factors all at once. Researchers may have concerns that the MGRS was also not able to account for or control for all the relevant social factors in the original development of the WHO Standards, but the key is that all MGRS participants around the world met the same predetermined criteria. These criteria were, at the time, the most important determinants of child growth that were possible to measure or to proxy for. Updates to the WHO Standards could incorporate additional factors based on the social determinants of health and nutritional status, such as food prices, access to high-quality diets, and food safety practices.

### Disentangling the Clinical and Epidemiological Meanings of Stunting

In this section, we describe the differences between the clinical and epidemiological meanings of growth faltering and summarize some articles in which the authors misapplied these concepts to argue for locally specific growth curves. By itself, an indication of stunting for an individual child at a point in time has limited clinical usefulness, and reductions in stunting prevalence would come more readily from improving social, economic, and environmental determinants, compared with changing standard clinical care.^27^ How a child grows over time is much more informative about their health than their length or height at a snapshot in time. Other measures of nutritional status, such as biochemical indicators, clinical indicators, and dietary indicators, must be used before making any nutrition or medical diagnoses.

Individual trajectories matter more than moments in time from a clinical perspective, but the choice of which growth curve to use in a clinical setting does matter for developing targets and interventions during a given clinic visit. The target for an individual child could differ depending on the growth curve used for reference, and this target informs what nutritional and medical interventions may be needed. Instead of developing locally specific growth curves, the focus should be on 1) gathering accurate growth information in clinical and research settings, 2) employing best practices in nutrition assessment including but not limited to anthropometric assessment, and 3) developing targets for catch-up growth, and 4) having regular follow-up with patients at key intervals.

Concerns with “misclassification” conflates clinical with epidemiological relevance. Stunting is a statistically constructed indicator, not a biological phenomenon.^7^^,^^47^ By construction of the HAZ, 2.3% of children in a healthy population would be labeled having stunting.^48^ An incorrect interpretation of stunting as a biological phenomenon instead of a statistical phenomenon also positions children who happen to be shorter as automatically less healthy, instead of that some individuals just happen to fall in the left tail of the HAZ distribution.^29^ The binary classification of children as having stunting or not may also detract attention from less severe degrees of linear growth faltering that are also negatively associated with child health outcomes.^48^^,^^49^

In the published literature that argues for locally specific growth curves, the clinical and epidemiological meanings of growth faltering are conflated. For example, Natale and Rajagopalan^50^ claimed that children may be misclassified as having stunting or not unless a locally specific growth curve were used and, therefore, that appropriate clinical strategies and policy would be misapplied. Hruschka^51^ argued that the WHO Standards will misclassify people as well nourished when they are not or undernourished when they are not. Hackman and Hruschka^52^ and Hruschka et al^53^ defined “basal” height-for-age as the minimum in situations of extreme deprivation and “accrued” height-for-age as the growth potential due to environmental factors that occur above basal height-for-age, an idea that is not supported by the evidence on intergenerational transmission of stunting over time. Roelants et al^54^ and Saari et al^55^ argued that because of secular trends in height, growth curves need to be updated regularly or else there is risk of misclassification of children; and Khadilkar^56^ argued that the WHO Standards lead to misclassification of children in low- and middle-income countries as stunted and wasted. These concerns with misclassification of children as stunted or wasted conflates the purpose of epidemiological data with clinical data. Epidemiological data describe the health of populations, whereas clinical data are about individual health. Misclassification of children during a clinical exam is clearly a problem, but epidemiological data need to classify the health of populations of children, and universal standards are needed to do this well and ethically.

No child should receive a nutrition or a medical diagnosis only with information from any growth chart, not even the WHO Standards. For clinicians or epidemiologists who are concerned about the accuracy of growth charts for a given population or for misclassifying children as stunted or wasted, such as Júlíusson et al^57^ for Belgian and Norwegian children, improving other aspects of nutritional assessment with the consultation of a dietitian or nutritionist would be useful. Relevant exceptions are for preterm infants and children with chronic health concerns such as cystic fibrosis, Down Syndrome, or renal disease, specialized growth *references* and clinical judgements from an interdisciplinary team are especially important for a full clinical nutrition assessment.^58^^,^^59^ Updates to growth references for children with chronic diseases that affect linear growth may lag behind certain medical advancements (eg, treatments) and new therapies by allied health fields which can help with growth, such as physical therapy (eg, building muscle) and speech therapy (eg, promoting safe swallowing of food).

### Distinguishing Between Growth Standards and Growth References

This section will explain the differences between growth references and growth standards and will summarize some articles which incorrectly conflate references and standards to argue for locally-specific growth curves. As we defined in the introduction, a *growth reference* describes the heights and weights of children in a population at a point in time, and a growth standard defines how children should grow. Children who grow in similar circumstances within a community would be expected to have a growth curve fit them more precisely if it were derived from their own local data compared to global data.

The difference between growth standards and references is a common misunderstanding, demonstrated for example in the title of a 2018 article,  which refers only to the “WHO reference.”^60^ Dwipoerwantoro et al^61^ Ziegler and Nelson^62^ and de Wilde et al,^63^ argued that local standards fit local children more accurately and therefore should be used instead of the WHO Standards.^61^^,^^62^ Another related tautology, exemplified in articles by Poh et al^64^ and Schaffrath Rosario et al,^65^ is that the WHO Standards are useful for international comparisons but that local references are more applicable to local populations. Other researchers claim that due to wide variation in height across populations, children should be compared with local populations.^57^^,^^66^^,^^67^

All researchers would prefer a more precise fit of their data to a model for easier analyses. Indeed, modeling growth outcomes relative to WHO Standards can result in either more or less within-population variation in a sample than really exists. If growth references fit the sample population better, statistical analyses that estimate the effects of an intervention will have better precision for that particular study and in that particular sample. The problem is that the children will not be compared with a healthy growth standard. Instead, they will be compared against one another in their own environments and cohort, which may have structural and systemic threats to their growth that would be lost in an analysis using a local growth reference.

Wanting to have better statistical precision in models and not being able to easily measure systemic threats to nutritional status are not a sufficient justification for arguing that there are biological differences in growth between different populations of children. The arguments for locally specific growth curves are tautological because a locally derived reference will fit a local population more precisely, by construction. Accurate and timely nutrition monitoring can and should be done using the WHO Standards, such as for Brazil, where several socioeconomic and health policies, as well as reductions in economic inequality, jointly contributed to huge declines in the prevalence of stunting from 37.1% to 7.1% between 1974 and 2007.^68^

Another potential source of deviation between any local references and the WHO Standards is simply due to misuse, for example, when clinicians mistakenly apply the chart to children born preterm, which happens commonly.^69^ Indeed, a review of articles found that differences in feeding practices and inclusion and exclusion criteria may explain many of the discrepancies between various growth curves.^70^ To avoid misclassification of children’s nutritional status, the main research efforts should be toward improving anthropometry and nutrition assessment.^7^

## CONCLUSION

Describing trends in child heights and weights over time is an important exercise because the nutrition transition continues to affect the distributions of body size at the community, national, and global levels.^71^ In addition, exploring inequities between racial or ethnic groups through stratification, when race or ethnicity is not being modeled as a determinant of nutritional status, is indeed necessary for informing resource allocation and for identifying health disparities. For example, in Guatemala, the finding that stunting was mainly caused by preventable social and environmental phenomena, including an “ecology of fear” from pervasive violence, helped spur national action toward combatting malnutrition in that country, in which child growth disparities are among the worst in the world.^21^^,^^72^ A universal approach to child growth using the WHO Standards prevents further marginalization of children who are particularly at risk for poor growth outcomes.

There are many scientific benefits of using one global standard for child growth around the world,^14^ and measuring children is necessary to identify those at risk for malnutrition.^73^ Representatives to a technical workshop on growth monitoring from 9 countries reported that measurements, lack of equipment, interpreting data, and counseling families were some key challenges that remain for ensuring accurate growth monitoring for their populations.^73^ These findings reflect similar challenges remaining from over 20 years ago found in survey data from 202 countries between 1998 and 2002.^74^ New technology and tools that combine growth monitoring efforts with immunization, child development, and social services show promise in many countries, and data quality for child heights and weights is improving.^73^

The scientific histories of anthropometrics and of statistics are intertwined, involving comparisons of body measurements among different races and ethnic groups. Researchers now have access to comprehensive anthropometric data from around the world, including from publicly available sources like the Demographic and Health Surveys, the Living Standards Measurement Study, and the UNICEF Multiple Indicator Cluster Surveys. Height, being a visible characteristic, is sometimes used to categorize people. However, arguing for different growth standards based on race or ethnicity assumes biological delineations between groups of people and overlooks the systemic causes of malnutrition, such as poverty. Researchers and clinicians must be cautious because regularly using locally specific growth curves may influence parental perceptions of child health. For example, the use of locally specific growth curves may deter parents seeking health care for their children because their children do not seem to be experiencing linear growth faltering, for younger children <5 years old as well as for older children and adolescents, for whom a growth standard does not exist. In their Figure 2, Shur et al^23^ describe the 1700 s and 1800 s as a “shameful past” of anthropometry as a field, but some of the aspects of that shameful past remain in the child growth literature today.

The push for alternate growth curves based on race, ethnicity, and nationality has been present for decades and still remains. Many researchers in nutrition and epidemiology work to address long-standing scientific racism in those fields; therefore, exploring the literature that argues for alternate growth curves is productive. Attributing deficits in growth to the diversity in race and ethnicity neglects the underlying social inequities, perpetuates hierarchical categorization, and aligns with scientific racism.^13^ The use of the WHO Standards and their future updates provide a standardized tool to monitor individual children in a clinical setting, screen children in a public health setting, and monitor populations.
